# Neural Tube Defects, Folic Acid and Methylation

**DOI:** 10.3390/ijerph10094352

**Published:** 2013-09-17

**Authors:** Apolline Imbard, Jean-François Benoist, Henk J. Blom

**Affiliations:** 1 Biochemistry-Hormonology Laboratory, Robert Debré Hospital, APHP, 48 bd Serrurier, Paris 75019, France; E-Mail: jean-francois.benoist@rdb.aphp.fr; 2 Metabolic Unit, Department of Clinical Chemistry, VU Free University Medical Center, De Boelelaan 1117, Amsterdam 1081 HV, The Netherlands; E-Mail: h.blom@vumc.nl

**Keywords:** neural tube defects, folate, methylation, choline, methionine, homocysteine, MTHFR, B12 vitamin

## Abstract

Neural tube defects (NTDs) are common complex congenital malformations resulting from failure of the neural tube closure during embryogenesis. It is established that folic acid supplementation decreases the prevalence of NTDs, which has led to national public health policies regarding folic acid. To date, animal studies have not provided sufficient information to establish the metabolic and/or genomic mechanism(s) underlying human folic acid responsiveness in NTDs. However, several lines of evidence suggest that not only folates but also choline, B12 and methylation metabolisms are involved in NTDs. Decreased B12 vitamin and increased total choline or homocysteine in maternal blood have been shown to be associated with increased NTDs risk. Several polymorphisms of genes involved in these pathways have also been implicated in risk of development of NTDs. This raises the question whether supplementation with B12 vitamin, betaine or other methylation donors in addition to folic acid periconceptional supplementation will further reduce NTD risk. The objective of this article is to review the role of methylation metabolism in the onset of neural tube defects.

## 1. Introduction

Neural tube defects (NTDs) are common complex congenital malformations of the central nervous system resulting from failure of the neural tube closure during embryogenesis. The prevalence of NTDs varies widely between 1 and 10 per 1,000 births, depending on geographic region and ethnical grouping, making them one of the most frequent congenital malformations [1].

NTDs can be classified in “open” NTDs in which the neural tissue is exposed and “closed” NTDs with the neural tissue covered by tissue [2,3]. “Open” NTDs include craniorachischisis resulting from a total failure of neurulation with most of the brain and the entire spinal cord remaining open, anencephaly when the defect occurs in the cranial region and spina bifida cystica when the defect is localized in the lumbosacral area. In this last defect, if only meninges and cerebrospinal fluid herniates through the defect, it is referred as meningocele, while a myelomeningocele directly involves spinal cord and/or nerve roots. “Closed” NTDs, encompass encephalocele and spina bifida occulta. Encephalocele is a defect of the bony skull through which part of the brain herniates. Spina bifida occulta, results from a gap in one or more vertebral arches in the lumbosacral area, but the spinal cord and meninges remain entirely within the vertebral canal. In these types of defects the folds may have come together, but the normal fusion process was disrupted.

Unlike the cranial defects, which are usually lethal at or before birth, spina bifida is compatible with postnatal survival. Spina bifida cystica is more severe; these patients being at increased risk for morbidity and mortality throughout their life. Only 1% of children born with an open NTD are free from disability. Affected patients usually have anesthesia of the skin, abnormalities of the hips, knees, and feet, reduced ability to walk or need a wheelchair, have little or no bowel and/or bladder control, and require frequent surgical interventions to minimize the effects of hydrocephalus [4]. Thus, the lifetime medical costs of spina bifida affected patients are considerable [5,6]. Finally, spina bifida occulta is the mildest form of spina bifida and is compatible with normal life.

NTDs have complex and multifactorial etiologies in which both genetic, life style and environmental factors appear to be involved. Chromosomal anomalies can be associated with NTDs, but represent only 2% to 16% of isolated NTDs [7]. Several observations support the view that genetic factors are involved in NTDs formation: first, an increased risk in some ethnic groups (e.g., Irish and Mexican) and second, the high recurrence risk for siblings of affected individuals [1,8,9]. In addition to genetic factors, environmental influences such as parental occupation, maternal obesity, and maternal nutritional status have been related to NTDs [1,2,8]. Particularly, it has been suggested more than 40 years ago that maternal folate status is associated with NTD risk [10]. A substantial number of reviews have been published on NTDs and folic acid [11,12,13,14,15,16]. Over the years, more and more studies suggest that not only folate but whole methylation metabolism could be involved in the etiology of NTDs. This review will focus on the involvement of methylation metabolism in the onset of neural tube defects.

## 2. Neurulation and Neural Tube Defects

Neurulation is a fundamental event in embryogenesis that culminates in the formation of the neural tube, which is the precursor of the brain and spinal cord (reviewed in [17,18,19]). Neurulation begins with the formation of the neural plate as a thickening of the dorsal ectoderm. Neural plate then shapes, with the processes including convergent extension. Then the neural plate bends, elevates and begins to move towards the midline. The extremities come into contact and fuse to create the neural tube, which, thereafter, becomes covered by epidermal ectoderm. Closure of the cranial neural tube is essential not only for maintenance of brain development but also for initial formation of much of the skull [20]. The fusion of neural folds, is subject to debate concerning the number of initiation sites of fusion and their location [21]. Neural tube closure depends upon the cooperation of several mechanisms: convergent extension of the neural plate, neuroepithelial apoptosis, neural crest cell migration, proliferation and differentiation. The development and closure of the neural tube is completed 28 days after conception. If neural tube closure fails, the embryo develops an NTD. However, some authors also support the possibility of some NTD resulting from a closed neural tube secondarily reopening [21].

## 3. NTD Prevention via Folate

### 3.1. The Folate Story

Since the 18th century, a relationship between social classes and prevalence of NTDs has been noted [22]. Lower social classes tend to have decreased concentrations of various vitamins, including folate, as a consequence of poor diet [23]. In the 60s it was suggested that low folate status was one specific risk factor for NTD by the Hibbard and Emery groups [10,24,25]. In a series of non-randomized cases/controls trials Smithells and coworkers showed that periconceptional supplementation with multivitamin containing 0.36 mg of folic acid (FA) reduced the recurrence rate of NTDs from 5.9% to 0.5% [26,27,28,29]. Although strongly suggestive, these reports were not regarded as definitive proof because the assignment to treatment groups was not randomized and because FA was given as a part of a multivitamin supplement.

In parallel, biological arguments have suggested that insufficient folate intake was related to NTDs. Although several studies failed to reach significance [30,31,32,33,34,35,36], most have found lower plasma folates [37,38,39,40], red blood cells (RBC) folates [37,38,39,41] or amniotic fluid (AF) folates [42] in women carrying NTD affected fetuses, or who gave birth to NTD affected offsprings, compared to control pregnant women. Interestingly, folates concentrations, even if decreased, remained generally within reference ranges.

A substantial number of cases/controls studies followed those from Smithells and coworkers (reviewed in [43]) and explored the relation between administration of folates and NTDs (recurrence) risk. One performed in Cuba with 5 mg/day of FA alone showed a recurrence rate dropping from 3.5% to 0% [44]. Others [45,46,47,48,49,50,51] have shown evidence of decreased NTD occurrence ranging between 35% to 75%, associated with FA containing multivitamin and high food folate consumption. In contrast, few studies did not detect a significant effect from dietary folate or multivitamin supplements on NTDs occurrence [52,53].

In parallel, Laurence *et al.* reported in 1981 the first double-blind randomized controlled trial for the use of FA in the periconceptional period and assessed for the first time that 2 mg/day of FA could prevent recurrence of NTDs [54]. However, the methodology used was criticized due to the small number of women included and the *a posteriori* change of two women with NTDs affected fetuses from the FA group to the non-compliers group [55]. In 1991, the Medical Research Council of the United Kingdom (MRC-UK) published the results from a large multicenter double-blind randomized trial [56]. This study is to date the largest and most important randomized trial comparing the effect of 4 mg/day FA *versus* multivitamin supplement (with or without FA) and *versus* placebo in a large cohort of women that had antecedents of NTD-affected children. The FA supplemented group (with or without multivitamins) had a decrease of 72% in the number of NTDs compared to placebo, while the multivitamin supplement without folic acid did not showed any significant protective effect. These results were decisive in demonstrating the specific preventive effect of FA on the recurrence risk of NTDs. The MRC trial was shortly followed by the largest occurrence risk study performed by Czeizel and Dudas, referred as the “Budapest trial” [57]. This randomized, large-scale trial compared the NTDs prevalence in women receiving 0.8 mg/d of FA *versus* trace element supplements. The results demonstrate a complete prevention of NTDs with no occurrence in the FA group among 2,104 women compared to six NTDs cases among 2,052 women in the control group. More recently, a large interventional study performed in China [58] demonstrated a lower occurrence rate with the use of 0.4 mg FA in the periconceptional period. This study also points out a greater reduction in NTD risk in areas with high baseline rates.

The MRC and Budapest trials, together with the China interventional study, undoubtedly showed a reduction in the occurrence and recurrence of NTDs with periconceptional FA supplementation, which has influenced public health policy, but the non-homogeneity of the doses of FA used in these trials makes it difficult to assess the dose of FA supplement that should be recommended in the prevention of NTDs.

### 3.2. National Programs for NTDs Prevention

**Figure 1:**
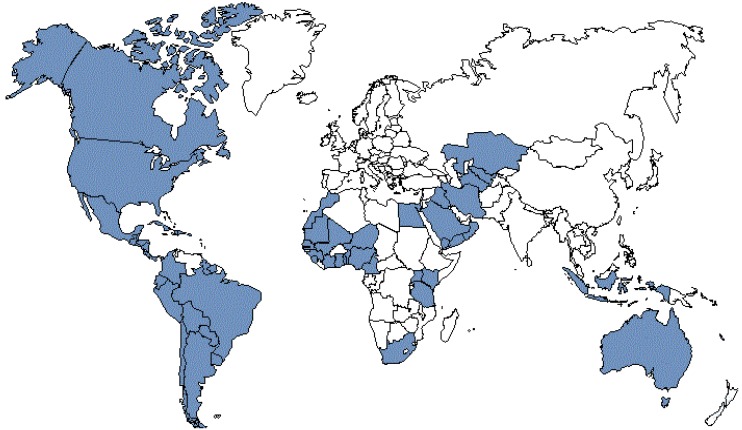
Countries with mandatory folic acid food fortification, adapted from [71].

There are three potential approaches for the delivery of folic acid to the general population: improvement of dietary habits, fortification of food and use of supplements [59]. In 1992 the CDC recommended the use of periconceptional FA supplementation of 4 mg per day for high risk women and 0.4 mg/day for all others [59]. Following these recommendations, Stevenson *et al.* observed a decline in NTD prevalence in USA [60]. In 1996, the Food and Drug Administration authorized food fortification with FA in the USA and this became mandatory in 1998 [61]. Grains and cereals were enriched with FA at a concentration of 1.4 mg/kg in order to increase the average consumption of women of reproductive age by around 0.1 mg per day. Many other countries including all North America countries, Australia and the majority of South America countries established similar policies (Figure 1).

Food fortification significantly increased serum and RBC folates among childbearing-aged women and decreased the prevalence of low folate concentrations [62,63,64,65,66]. A “positive side effect” was also observed with fortification: the decrease of plasma total homocysteine concentrations in the population, a cardiovascular risk factor [67,68]. Numerous studies have evaluated the prevalence of NTDs before and after food fortification, and the results are summarized in Table 1. Most of them show a decrease between 10% and 80% in the total NTD prevalence associated with the introduction of food FA fortification. Moreover, the decline in NTD was most obvious in areas [69] or ethnic groups with high baseline prevalence [70].

**Table 1:** Reduction in NTD rates with folic acid fortification.

Reference	Area/State	Period studied		Total NTD prevalence rate (/1,000 births)		% Reduction
		Before fortification	After fortification	Before fortification	After fortification	
Ray *et al*. [72]	Ontario	1994–1997	1998–2000	1.13	0.58	49
Honein *et al*. [73]	USA	1995–1996	1998–1999	0.38	0.31	19
Persad *et al*. [74]	Nova Scotia	1991–1997	1998–2000	2.58	1.17	55
Williams *et al*. [75]	USA	1995–1996	1998–1999	0.76	0.56	26
De Wals *et al*. [76]	Quebec	1992–1997	1998–2000	1.89	1.28	32
Palomaki *et al*. [77]	Maine	1993–1996	1998–2000	1.23	1.07	13
Lambert-Messerlian *et al*. [78]	Rhode Island	1991–1996	1998–2000	3.8	3.3	13
Simmons *et al*. [79]	Arkansas	1993–1995	1999–2000	1.09	0.82	25
Liu *et al*. [64]	Newfoudland	1991–1997	1998–2001	4.36	0.96	78
Chen *et al*. [80]	Costa Rica	1996–1998	1999–2000	9.7	6.3	35
Hertrampf *et al*. [81]	Chile	1999–2000	2001–2002	1.70	1.01	41
Lopez-Camelo *et al*. [82]	Chile	1982–1991	2001–2002	1.57	0.80	49
Canfield *et al*. [83]	USA	1995–1996	1999–2000	0.71	0.5	30
De Wals *et al*. [69]	Canada	1993–1997	2000–2002	1.58	0.86	46
Chen *et al*. [84]	California	1989–1996	1998–2003	0.59	0.70	No decline
Sayed *et a*l. [85]	South Africa	2003–2004	2004–2005	1.41	0.98	31
Amarin *et al*. [86]	Jordan	2000–2001	2005–2006	1.85	1.07	49

Only one study [84] did not observe any effect of fortification although this study highlights a continuous decreasing prevalence of NTD before FA food fortification. This emphasizes the difficulty in interpreting these results because numerous other factors such as life-style changes, ethnic background drift or socio-economic conditions can influence NTDs risk. Other confounding factors, such as increased prenatal diagnosis and concomitant termination of NTD affected pregnancies will also have influenced the results. Nevertheless, the sum of all these studies provides evidence that folic acid fortification decreased the incidence of NTDs. Finally, economic analyses have shown that food fortification was a success in terms of costs/benefit ratio [6,87,88].

**Figure 2:**
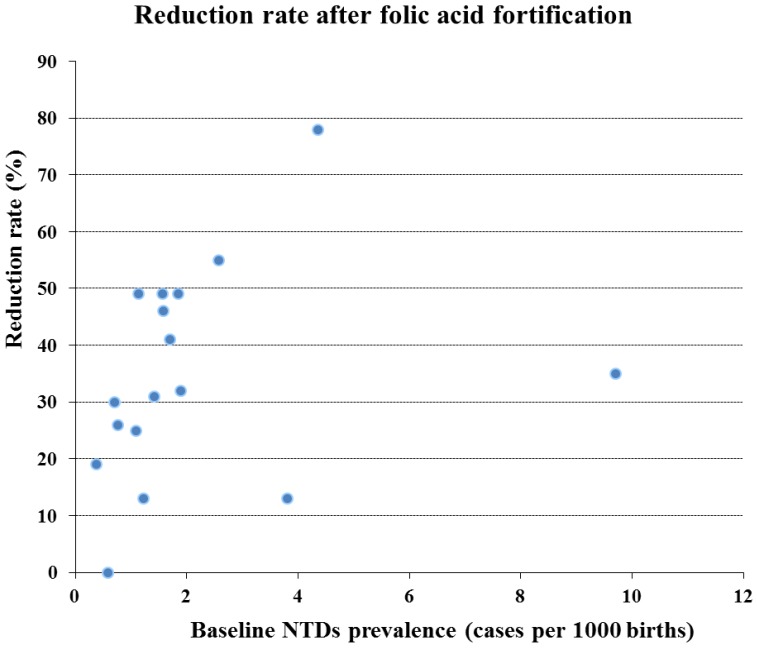
Reduction rate of NTDs after FA food fortification depending on baseline prevalence.

Despite its efficacy, two issues are still a matter of debate regarding food fortification: the appropriate dose to use and potential side effects [89]. Several authors argued that the current amount of FA might be too low to prevent all folic-acid “sensitive” NTDs and favor to increase the FA dose [90,91,92,93]. Their logic is supported by the fact that there seems to be a dose response effect in NTD prevention related to folate supplementation. In fact, NTD rates seem to inversely correlated with the amount of folate intake and/or with maternal RBC and serum folate concentrations [66,94,95,96]. On the other hand, probably not all NTDs cases are sensitive to FA. Reduction of NTDs prevalence after FA food fortification seems to be correlated with NTD baseline prevalence (Figure 2). It remains a challenge to establish whether the number of FA preventable and FA non-preventable NTD cases [97,98]. When Figure 2 is extrapolated it can be deduced that folic acid food fortification will not reduce the number of NTDs below about 0.5 per 1,000 births.

Possible side effects of high dose of FA are still being debated (reviewed in [99]). Historically, concerns surrounding FA use have focused on the possibility that folic acid could mask pernicious anemia caused by B12 deficiency [100]. However, it is now recognized that the diagnosis of pernicious anemia should be based on serum B12 measurements, which are not influenced by folates, and not only on haemoglobin concentration or haematocrit. Recently, focus has shifted to the possibility that FA intake might lead to epigenetic changes and concerns about cancer development and outgrowth [89,101]. However, a very recent meta-analysis of the effects of B vitamins has not shown that there is an increased risk of cancer incidence [102]. Moreover, since the implementation of mandatory folic acid fortification in the United States in 1998 both incidence and mortality of colorectal cancer have continued to decline [103]. These observations, led the European Food safety authority to report that the current evidence does not show an association between high folic acid intakes and cancer risk but they were also unable to confidently exclude such a risk [89]. Finally, some animal studies using FA supplementation reported unexpected side effects of FA on embryonic development [104,105]. Pickell *et al.* observed embryonic delay and growth retardation using high doses of FA in pregnant mice. In their study, Marean *et al.* used short- or long-term FA enriched diet at doses comparable with those used in food fortification and observed increased NTD rates in some mouse models of non-FA responsive NTDs.

In the non-fortified countries, including the whole of Europe, health policies vary from no recommendations to dietary recommendations with or without supplements either for selected at-risk women or all women of childbearing age [106,107]. Despite these actions, clear decreases in NTDs rates have not been seen in all these countries [106,107,108,109]. The awareness of the benefits of folic acid supplementation improved with health campaigns [110] but many women, especially in their first pregnancy, are still unaware that folic acid must be taken before conception [107,111,112,113]. Ray *et al.* reviewed the prevalence of folic acid supplementation in community programs worldwide and found that associations with low uptake were unintentional pregnancies, young age and low income mothers [114]. As such, some authors are arguing for food fortification while others argue for more health education campaigns [115,116,117,118].

## 4. Folate and Methylation Metabolism

Folate is an essential water soluble B-vitamin provided in the diet, particularly from fruits and vegetables. In general, the term folic acid is applied to the more stable synthetic form while the term folate referred to the natural forms. Natural dietary folates consist mainly of 5-methyltetrahydrofolate (5-MeTHF) and 10-formyltetrahydrofolate (10-formylTHF) in their polyglutamated forms. These consist of one to six glutamate molecules joined in a peptide linkage. In the gut polyglutamates are required to be hydrolyzed into monoglutamates to be absorbed [119,120]. Folic acid has only one glutamic residue and first needs to be reduced to the naturally bioactive form tetrahydrofolate (THF), through two reduction reactions catalyzed by dihydrofolate reductase. The addition of a one-carbon unit and subsequent reduction steps produce 5-MeTHF-monoglutamate, which is the main circulating form in blood [12]. 5-MeTHF is taken up in to the cells by tissues specific folate receptors or carriers proteins, where they are accumulated after their transformation into polyglutamates. Polyglutamated folates do not cross membranes and are in this way retained in the cell. They also have a higher affinity for enzymes involved in folate metabolism than their monoglutamates counterparts.

In cell (Figure 2), 5-methylTHF can donate a methyl-group for homocysteine remethylation to methionine by methionine synthase. The resulting THF can directly be converted into 5,10-methylene THF by the action of serine hydroxymethyltransferase, which has a cytosolic as well as a mitochondrial isoform. THF can also be converted in 5,10-methyleneTHF (5,10-MTHF), via 10-formylTHF and 5,10-methenylTHF catalyzed by the trifunctional enzyme methylene-tetrahydrofolate dehydrogenase (MTHFD1) that has formyltetrahydrofolate synthetase, methenyl-tetrahydrofolate cyclohydrolase and methylenetetrahydrofolate dehydrogenase activity. Tetrahydrofolate synthetase activity is also present in the mitochondrion, in the form of the monofunctional MTHFD1L enzyme. 5,10-MTHF can be reduced to 5-MeTHF by the riboflavin (vitamin B2) dependent enzyme methylenetetrahydrofolate reductase (MTHFR).

Mitochondria contain around 40% of total cellular folates, and particularly, folates resulting from the catabolism of dimethylglycine, sarcosine and glycine. Polyglutamated folates in mitochondria and cytoplasm constituted two distinct pools that are not directly in equilibrium through folates exchange. The exchange of formate, a 1-C unit equivalent between the two compartments contributes indirectly to this equilibrium [121,122].

In cytoplasm, folates are involved in three principal metabolic pathways that comprise: homocysteine remethylation, *de novo* purine biosynthesis and *de novo* dTMP biosynthesis. *De novo* synthesis of dTMP from deoxyuridylate involves the enzyme thymidylate synthase which is in competition with MTHFR for the cytoplasmic 5,10-MTHF. *De novo* purine nucleotide biosynthesis is a 10-step pathway that uses 10-formylTHF to supply the number 2 and 5,10-methenyl-THF to supply the number 8 carbons of the purine ring.

**Figure 3:**
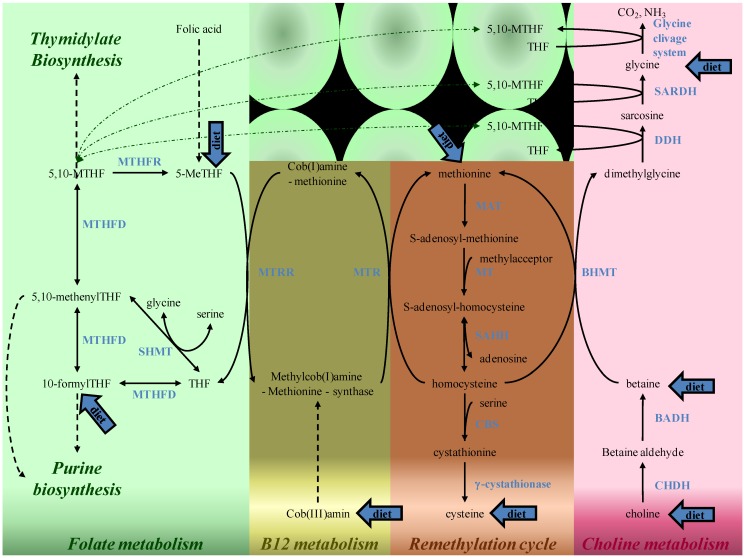
Simplified folate metabolism and its interrelation with the remethylation cycle, vitamin B12 and choline metabolism. Enzymes are in blue. Blue arrows indicate metabolites that can be provided by the diet. BADH, betaine aldehyde dehydrogenase; BHMT, betaine-homocysteine methyltransferase; CBS, cystathionine β-synthase; CHDH, choline dehydrogenase; DDH, dimethylglycine dehydrogenase; MAT, methionine adenosyltransferase; MT, methyltransferases; MTHFR, methylenetetrahydrofolate reductase; MTHFD, methylenetetrahydrofolate dehydrogenase/methylenetetrahydrofolate cyclohydrolase/formyltetrahydrofolate synthetase; 5-MeTHF, 5-methyltetrahydrofolate; 5,10-MTHF, 5,10-methylenetetrahydrofolate; MTR, methionine synthase; MTRR, methionine synthase reductase; SAHH, S-adenosylhomocysteine hydrolase; SARDH, sarcosine dehydrogenase; SHMT, serine hydroxymethyltransferase; THF, tetrahydrofolate.

In the remethylation pathway folates closely relate to betaine, choline and cobalamin metabolisms [123,124,125] (Figure 3). Likely, in all tissues except red blood cells, homocysteine can be remethylated by using 5-MeTHF as methyldonor for the ubiquitous methionine synthase which requires cobalamin as a cofactor. The essential amino acid methionine can subsequently be converted in S-adenosyl-methionine, also called the universal methyl donor. S-adenosyl-methionine is the substrate for almost all methylation reactions in mammals such as methylation of proteins, nucleic acids, lipids, neurotransmitters and creatine synthesis [126]. Upon transfer of the methyl group, S-adenosyl-methionine is converted to S-adenosyl-homocysteine, which is hydrolyzed by SAH hydrolase (SAHH) to adenosine and homocysteine. If inadequate amounts of 5-MeTHF or cobalamin are available, homocysteine accumulates. Because the equilibrium of the SAHH reaction favours S-adenosyl-homocysteine formation, an accumulation of homocysteine can lead to the buildup of S-adenosylhomocysteine, a potent inhibitor of numerous methyltransferases. In specific tissues (kidney and liver), homocysteine can also be remethylated by betaine-homocysteine methyl-transferase (BHMT) using betaine. Betaine is provided in the diet and, probably in higher quantities from choline catabolism. Choline itself can be provided either in the diet or by two biosynthesis pathways which act through the methylation of phosphatidylethanolamine or through the CDP-choline pathway. Finally, homocysteine can be eliminated via the transulfuration pathway into cysteine. 

## 5. Putative Mechanisms of Folates Action

Folates play a key role in NTD susceptibility however it is noteworthy that maternal levels in most affected pregnancies are still within the “normal” range. Animals fed with inadequate folic acid amounts leading to significant reduction in maternal circulating folates and increased homocysteine levels do not result in NTDs nor an apparent effect on embryonic folates content [127,128]. On the other hand, embryos from these mothers showed intrauterine growth retardation [128]. As such, it is likely that profound maternal folate deficiency is not sufficient to cause NTDs.

It has been suggested that specific folates deficiency at the cellular level may be responsible for NTDs due to disturbed folates bioavailability. In fact, autoantibodies binding folate receptors and blocking the cellular uptake of folates have been described more frequently in women with NTDs affected fetuses [129]. In mice, inactivation of the gene *FOLR1* coding for a protein involved in folate transport in neuroepithelial, neural crest and visceral endoderm cells results in neural tube, heart and cranial structure malformations [130,131,132]. Supplementing heterozygous *FOLR1* knock-out mice with FA rescued the embryos in a dose-dependent manner [133]. Conversely, inactivation of the more expressed folate transporter *FOLR2* and heterozygous knock-out mice of the ubiquitous transmembrane transporter *RFC1* resulted in no neural abnormalities (homozygous knock-out is lethal) [131,134]. Consequently, the effect of FA supplementation could be to overcome the increased folate requirement of neuroepithelial cells by increasing folate bioavailability.

Folates may be related to NTD risk through their roles in nucleotide synthesis. In fact, in embryos, the rapidly dividing cells of the developing neural tube require the synthesis of large amounts of nucleotides in order to facilitate DNA replication. Barber *et al*. proposed the hypothesis that if neuroepithelial cells do not have an adequate internal supply of nucleotides, cellular replication will slow down and the development of the neural folds will be retarded [135]. This hypothesis is supported by the Splotch embryos mouse model, which demonstrates a homozygous loss of function mutation in the *Pax3* gene. In this model of FA responsive NTDs a deficiency of dTMP biosynthesis has been observed [136]. NTDs in the Splotch model can be rescued by either folic acid [136,137] or thymidine supplementation [136]. This finding suggests that folic acid prevents NTDs by rescuing *de novo* thymidylate biosynthesis. At the cellular level, the supplementation with FA has an effect on the differentiation, proliferation, and junction formation in neurosphere from splotch embryos that could explain the FA effect on NTD in this model [138]. Impaired thymidylate biosynthesis has also been suggested in another mouse model. In the *SHMT1* null mice, embryos exhibit exencephaly consequently to maternal folate deficient diet [139,140]. In fact, SHMT1 is involved in the repartition of 1-C unit between thymidylate biosynthesis and homocysteine remethylation. In this mouse model, a potential impaired thymidylate biosynthesis is responsible for NTDs only when associated with maternal folate deficiency. Finally, in the recent *MTHFD1* homozygous knock-out mice, which have impaired *de novo* purine synthesis, neural tube closure was abnormal and embryos died prematurely before E10.5. No NTD was observed in heterozygous mice. Further investigations are required in this model [141]. These three animal models suggest a basis for the role of folates in NTD prevention as a correcting factor for limited nucleotides biosynthesis. Other mechanisms also play a role because in another FA responsive NTD mouse model, the *Cited2* deficient mice, thymidylate biosynthesis was not different from wildtype animals [142].

As folates are also involved in the methylation pathway, several authors suggest that disturbed methylation could be responsible for the relation between folate and NTDs. In two studies, Dunlevy *et al*. disrupted the methylation cycle in cultured mouse embryos with ethionine and cycloleucine or methionine administration during the period of cranial neurulation. This resulted in a specific increased prevalence of NTDs without generalized toxicity such as growth retardation or developmental delay. They conclude that adequate functioning of the methylation cycle is essential for cranial neural tube closure in the mouse [143,144]. In another model, Afman *et al*. observed a delay in the neural tube closure in chick embryos treated with inhibitors of the methylation cycle [145]. Finally, in the *Axd* and the *Amt* mouse mutants, being NTD mouse models unresponsive to FA, high doses of methionine given during embryogenesis reduced NTD incidence [146,147]. The gene involved in the *Axd* mouse model has not yet been identified however the response to methionine suggests the involvement of methylation metabolism in this NTD mouse model. The *Amt* mouse model is a knock-out for a gene included in the mitochondrial glycine cleavage complex. The inactivation could thus induce a diminution of the mitochondrial and possibly total cellular folates pool leading to decreased bioavailability of 5-MeTHF for the methylation pathway.

The cellular, molecular or genetic mechanisms by which methylation interfere with neurulation are still largely unknown. Disturbance of the methylation cycle leads to decreased S-adenosylmethionine, increased S-adenosylhomocysteine or decreased S-adenosylmethionine/S-adenosylhomocysteine ratio, all leading to reduced cellular methylation capacity. As previously stated, S-adenosyl methionine is the methyl donor for numerous reactions including protein, lipid, and DNA methylation. On the other hand S-adenosyl-homocysteine is a potent inhibitor of most methyltransferases [126]. One study [148] has suggested that abnormal DNA methylation secondary to remethylation metabolism abnormalities could be involved in the etiology of NTDs. Significant hypomethylation of genomic DNA in the brain have been found in NTDs affected fetuses [149]. Homozygous null embryos for *DNMT3B70*, coding for one of the enzymes for DNA methylation is responsible for cranial NTDs [150]. Dysregulation of gene expression has also been observed in chick embryos, treated with methylation inhibitors leading to a widening of the anterior neuropore [151]. On the other hand, NTDs are not observed in null embryos for *MTHFR*, despite a significant reduction in the methylation ratio and global DNA methylation [152]. Bjorklund *et al*. hypothesized that post translational methylation of proteins from the cytoskeleton required for neural tissue differentiation could be involved in NTDs etiology [153]. In agreement with this hypothesis, methionine has been seen to influence the cytoplasmic distribution of actin and tubulin in neuroepithelial cells during neural tube closure [154].

## 6. Arguments for the Implication of Homocysteine, Vitamin B12 and Choline Pathways

### 6.1. Biological and Nutritional Aspects

#### 6.1.1. Homocysteine

Increased total homocysteine has been found in plasma [37,40,155,156,157,158,159] and in amniotic fluid [34,160] of women with NTD affected fetuses or with a history of NTD affected fetuses although these results are inconsistent [34,35,38,161,162,163,164,165,166]. Increased S-adenosylhomocysteine has also been noted in plasma of women with a history of NTD [40,166]. This results in a decreased S-adenosylmethionine/S-adenosylhomocysteine ratio. Finally, reports have related increased maternal methionine intake with decreased NTD risk [167,168,169]. Taken together, these results suggest that a disturbed remethylation cycle could be involved in the etiology of NTDs. The influence of methionine intakes and methionine concentrations requires further study.

Levels of homocysteine, increase when the remethylation cycle is disturbed which could itself be toxic. Among the possible mechanisms of homocysteine toxicity, some authors have suggested that homocysteine can act on N-methyl-D-aspartate (NMDA) receptors [170]. Others have hypothesized that homocysteine could have an effect through the increased homocysteinylation of proteins [132]. Several studies in animals have examined the embryotoxicity of homocysteine with conflicting results (reviewed in [171]). Afman *et al*., have shown a dose dependent closure delay of the anterior neuropore in chick embryos with homocysteine administration [172]. In a comparable experiment, Rosenquist *et al*. found increased NTDs in mouse embryos treated with homocysteine [173]. In rat embryos, Vanaerts *et al*. have shown a protective effect of MeTHF, against homocysteine embryotoxicity [174]. 

However, a relationship between homocysteine and neural tube closure was not observed by Greene *et al*. and Bennett *et al*., who did not show increased incidence of NTDs in mouse embryos associated with homocysteine exposure [175,176]. Consistent with these findings, the incidence of NTDs is not increased in mice models of hyperhomocysteinemia due to knocking-out of the *CBS* or *MTHFR* genes [152,177].

#### 6.1.2. Vitamin B12

As for folates, involvement of vitamin B12 in NTD risk has been suggested more than 30 years ago [33]. Recently, Carmichael *et al*. have shown that a high maternal intake of vitamin B12 is associated with decreased NTD risk [178]. Decreased vitamin B12 [30,31,35,36,42,179] has been found in AFs from women carrying NTDs affected fetuses.

However, data on the impact of maternal blood concentration of B12 are conflicting. Vitamin B12 concentrations were investigated in maternal blood of women carrying a NTD affected fetus or women with a history of NTD affected fetus (reviewed in [180]). Some of these studies have found lower B12 concentrations in these mothers compared to controls [38,156,157,162,164,181,182,183,184,185,186] while others do not reach statistical significance [30,32,40,159,161,163,165,187,188,189,190,191]. Two meta-analysis have identified an association between low maternal serum vitamin B12 and the risk of NTDs [192,193].

Vitamin B12 bound to transcobalamin (holo-TC) represents the pool of vitamin B12 available for uptake by cells and may represent a better marker of the bioavailability of vitamin B12. Only few studies have measured holo-TC levels in the serum of women with current or previous NTD affected pregnancies and they all found an increased risk of NTDs associated with lower levels of holo-TC [187,194,195].

Elevated methylmalonic acid is a metabolic marker of vitamin B12 insufficiency and is considered a better marker for vitamin B12 deficiency than the measurement of vitamin B12 itself. However, there are too few data to evaluate MMA as a risk factor for NTDs [188,196]. Taken together, these data form a body of evidence for the role of vitamin B12 in the etiology of NTD. Whether B12 administration will prevent NTDs has not been studied yet.

#### 6.1.3. Choline and Betaine

The role of choline and betaine, which are alternative methyl donors for 5-MeTHF in homocysteine remethylation via betaine-homocysteine methyltransferase, have been investigated in NTDs. The relationship between periconceptional choline and betaine intake and NTD was first investigated by Shaw *et al*. in pregnant women [197]. Women in the highest quartile of choline intake had a risk reduction of about 50% compared with those in the lowest quartile. These results were not confirmed in more recent studies from the same group [178,198,199]. However, in Hispanic women a decreased NTD risk was associated with high betaine intakes when they stratified their results for ethnicity [178].

Shaw *et al*. measured total serum choline, which is mainly constituted by phosphatidylcholine, in pregnant women and found that lower choline was associated with an increased NTD risk in a folate fortified population [200]. For serum betaine no association was found.

The *in vitro* treatment of mouse embryos with choline uptake or choline metabolism inhibitors was responsible for developmental defects affecting the neural tube and the face [201]. These results suggest a putative role of choline metabolism in the etiology of some neural tube defects. More studies are needed to explore the potential role of these methyl donors in NTD risk.

### 6.2. Genetic Aspects

Kang *et al*. first described a thermolabile variant of the methyltetrahydrofolate reductase (MTHFR) which interfers with folate and remethylation pathways [202]. This variant is due to the 677C > T polymorphisms (Ala222Val, rs1801133) and was first associated with NTD risk by Van der Put *et al*. [203]. It leads to mild to highly increased plasma total homocysteine concentrations depending on the folate status. Numerous studies have looked at the relationship between NTD risk and genotypes of mothers with children with NTD or children affected with NTD; these are summarized in Table 2. Several meta-analyses have been performed and all have found a significantly increased risk of NTDs associated with *MTHFR* 677C > T either in cases and maternal and even paternal genotypes [12,204,205,206,207]. The strength of this association was probably influenced by the ethnic origin of the populations studied and the riboflavin as well as the folate status.

Following the identification of the *MTHFR* 677C > T variant, many potential polymorphisms in genes involved in folate, remethylation, B12 and choline pathways have been explored in NTDs (Table 2). Only few of them may be associated with NTDs risk: *MTHFD1*1958G > A (rs2236225), *MTHFD1L* (rs3832406), *MTHFR* 1298A > C (rs1801131), *reduced folate carrier A* (*RFCI*) 80 A > G (rs1051266) *MTR* 2756 A > G (rs1805087) and *MTRR* 66A > G (rs1801394).

Interpretation of these studies is complex. For example, the majority of genetic studies in children have been performed on live NTDs-affected children which are in general the mildest affected forms. This may introduce bias. Moreover, it seems that the association between polymorphisms and NTD cases can be influenced by other polymorphisms through gene-gene interactions [208,209,210,211], by biological or nutritional factors [211,212] or by folic acid status and supplementation [208,213,214]. The association between polymorphisms and NTDs risk might also be different according to the type or the localization of NTD [160,215,216].

## 7. Conclusions

The protective effect of folic acid on the occurrence and recurrence of NTDs has been clearly demonstrated 20 to 25 years ago. However, the mechanisms underlying folic acid responsive NTDs remains to be elucidated.

It seems that folate status alone is insufficient to cause NTD but that it interacts with multiple genetic and environmental factors that are individually insufficient to cause NTDs. Elegant examples of gene-environment interactions are the *SHMT1* and Sploch embryos mouse models in which a folate deficient diet increases NTD frequency [127].

**Table 2:** Polymorphisms from genes involved in folate, choline, B12 vitamin pathway and remethylation cycle that have been studied for their implication in NTDs risk.

	Gene	Polymorphism(s) studied	Population studied	Effect on NTD risk	Reference
B12 Pathway	CUBN	rs1907362	Children	Decreased risk	Franke *et al*. [217]
		rs4748353	Children	NS	Franke *et al*. [217]
	TCN2	rs1801198	Children	NS	Guéant-Rodriguez *et al*.; Afman *et al*.; Swanson *et al*. [218,219,220]
			Mothers	NS	Candito *et al*.; Afman *et al*.; Swanson *et al*. [37,218,219,220]
				Increased risk	Pietrzyk *et al*. [221]
		rs96067256 rs4820889 rs9621049 rs1131603	Mothers and Children	NS	Afman *et al*.; Swanson *et al*. [218,219,220]
Choline pathway	BHMT	rs3733890	Children	NS	Zhu *et al*.; Morin *et al*. [222,223]
			Mothers	NS	Morin *et al*. [222]
	BHMT2	rs626105	Children	NS	Zhu *et al*. [223]
	CHKA	hCV1562393C	Children	NS	Enaw *et al*. [199]
		rs1562388	Children	Decreased risk	Enaw *et al*. [199]
	PCYT1A	rs3772109	Children	NS	Enaw *et al*. [199]
	SARDH	rs573904	Children	Increased risk	Franke *et al*. [217]
Folate pathway	DHFR	19bp DEL	Fathers	NS	Johnson *et al*. [224]
		19bp DEL; 9bp repeat	Children	NS	Johnson *et al*.; Van der Linden *et al*.; Doudney *et al*. [224,225,226]
		19bp DEL; 9bp repeat	Mothers	NS	Johnson *et al*.; Van der Linden *et al*. [224,226]
	Receptor Folate	rs651646	Fathers	NS	Oleary *et al*. [227]
			Children	NS	Oleary *et al*. [227]
			Mothers	NS	Oleary *et al*. [227]
	GCPII	rs61886492	Children	NS	Relton *et al*. [228]
			Mothers	NS	Morin *et al*.; Relton *et al*. [228,229]
	MTHFD1	rs2236225	Fathers	NS	Brody *et al*.; De Marco *et al*. [230,231]
			Children	NS	Brody *et al*.; Hol *et al*.; Van der Linden *et al*.; Doudney *et al*. [225,226,230,232]
				Increased risk	De Marco *et al*. [231]
			Mothers	Increased risk	Brody *et al*.; De Marco *et al*.; Parle McDermott *et al*. [230,231,233]
				NS	Van der Linden *et al*. [234]
		rs1950902	Fathers	NS	Brody *et al*. [230]
			Children	NS	Brody *et al*. [230]
			Mothers	NS	Brody *et al*. [230]
	MTHFD1L	rs3832406	Children and mothers	Increased risk with “allele 1”	Parle Mc Dermott *et al*. [235]
	MTHFR	rs2066462	Fathers	NS	Morrison *et al*. [236]
			Children	NS	Morrison *et al*. [236]
			Mothers	NS	Morrison *et al*. [236]
		rs1801131	Fathers	NS	Van der Put *et al*.; Volcik *et al*.; De Marco *et al*.; Parle McDermott *et al*.; Boduroglu *et al*.; Grandone *et al*.; Gonzales-Herrera *et al*.; De Marco *et al*. [163,216,237,238,239,240,241,242]
				Increased risk	De Marco *et al*. [239]
			Children	NS	Van der Put *et al*.; Volcik *et al*.; Parle McDermott *et al*.; Relton *et al*.; Boduroglu *et al*.; Felix *et al*.; Grandone *et al*.; Gonzales-Herrera *et al*.; Doudney *et al*.; Behunova *et al*.; De Marco *et al*. [162,163,216,228,237,238,240,241,242,243]
				Increased risk	De Marco *et al*. [239]
			Mothers	NS	Van der Put *et al*.; Volcik *et al*.; Parle McDermott *et al*.; Gutierrez-Revilla *et al*.; Relton *et al*.; Boduroglu *et al*.; Grandone *et al*.; Felix *et al*.; De Marco *et al*. [162,163,216,228,237,238,241,242,244]
				Increased risk	De Marco *et al*.; Gonzales-Herrera *et al*.; Candito *et al*. [37,239,240]
	MTHFR	rs1801133	Fathers	NS	Papetrou *et al*.; Van der Put *et al*.; Morrison *et al*.; Van der Put *et al*.; Volcik *et al*.; Parle McDermott *et al*.; Rampersaud *et al*.; Boduroglu *et al*.; Grandone *et al*. [163,203,216,236,237,241,242,245,246]
				Increased risk	Blom *et al*. * [12]
			Children	NS	Papetrou *et al*.; Mornet *et al*.; Koch *et al*.; Morrison *et al*.; Shaw *et al*.;Van der Put *et al*. *; Christensen *et al*.; Volcik *et al*.; Gonzales-Herrera *et al*.; Gutierrez-Revilla *et al*.; Felix *et al*.; Boduroglu *et al*.; Doudney *et al*.; Behunova *et al*. [38,162,214,216,225,236,237,242,243,244,245,247,248,249]
				Increased risk	Whitehead *et al*.; Kirke *et al*., Ou *et al*.; Van der Put *et al*. *; De Franchis *et al*.; Shields *et al*.; Botto *et al*. *; Johanning *et al*.; Parle McDermott *et al*.; Johanning *et al*.; Pietrzyk *et al*.; Rampersaud *et al*.; Kirke *et al*.; Relton *et al*.; Blom *et al*. *; Grandone *et al*.; Wenstrom *et al*. [12,41,160,163,205,206,241,246,248,250,251,252,253,254,255,256,257]
				Decreased risk	Guéant-Rodriguez *et al*. [219]
			Mothers	NS	Papetrou *et al*.; Morrison *et al*.; Christensen *et al*.; Ubbink *et al*.; Lucock *et al*.; Volcik *et al*.; Gonzales-Herrera *et al*.; Parle McDermott *et al*., Rampersaud *et al*.; Gutierrez-Revilla *et al*.; Relton *et al*.; Boduroglu *et al*.; Felix *et al*.; Grandone *et al*.; Candito *et al*.; Shang *et al*.; Davalos *et al*. [37,38,162,163,191,215,216,228,236,237,241,244,245,246,247,258,259]
				Increased risk	Van der Put *et al*. *; Van der Put *et al*.; Botto *et al*. *; Martinez *et al*.; Pietrzyk *et al*.; Yan *et al*. *; Blom *et al*. *; Amorim *et al*. *; Richter *et al*. [12,204,205,206,207,252,255,260,261]
	RFCI	61bp repeat	Fathers	NS	Oleary *et al*. [262]
			Children	NS	Oleary *et al*. [262]
			Mothers	NS	Oleary *et al*. [262]
		rs1051266	Fathers	NS	De Marco *et al*.; Viera *et al*.; De Marco *et al*.; Pei *et al*.; Oleary *et al*. [209,238,262,263,264]
			Children	NS	De Marco *et al*.; Shaw *et al*.; Oleary *et al*.; Doudney *et al*.; Wang *et al*. *; Relton *et al*. [213,225,228,238,262,263,264,265]
				Increased risk	De Marco *et al*.; Pei *et al*. [209,263]
				Decreased risk	Franke *et al*. [217]
			Mothers	NS	De Marco *et al*.; De Marco *et al*.; Morin *et al*.; Oleary *et al*.; Wang *et al*. *; Relton *et al*. [209,228,229,238,262,265]
				Increased risk	Pei *et al*.; Shang *et al*. [215,263]
	SHMT	rs1979277	Children	NS	Heil *et al*.; Relton *et al*. [41,266]
			Mothers	NS	Heil *et al*.; Relton *et al*. [41,266]
		delTCTT1721_1724	Children	NS	Heil *et al*. [266]
			Mothers	NS	Heil *et al*. [266]
Remethylation pathway	MTR	rs1805087	Fathers	NS	Morrison *et al*.; Oleary *et al*.; De Marco *et al*. [236,239,267]
			Children	NS	Van der Put *et al*.; Morrison *et al*.; Shaw *et al*.; Johanning *et al*.; Zhu *et al*., Oleary *et al*.; Doudney *et al*.; De Marco *et al*.; Morrison *et al*. [225,238,251,267,268,269,270,271]
				Decreased risk	Christensen *et al*. [38]
				Increased risk	Guéant-Rodriguez *et al*. [219]
			Mothers	NS	Van der Put *et al*.; Morrison *et al*.; Christensen *et al*.; Doolin *et al*.; Zhu *et al*.; Oleary *et al*.; Candito *et al*.; Ouyang *et al*. *; De Marco *et al*. [37,38,236,239,267,270,271,272,273]
	MTRR	rs162036	Fathers	NS	Oleary *et al*. [267]
			Children	NS	Oleary *et al*. [267]
			Mothers	NS	Oleary *et al*. [267]
		rs1532268	Fathers	NS	Oleary *et al*. [267]
			Children	NS	Oleary *et al*. [267]
			Mothers	NS	Oleary *et al*. [267]
		rs1801394	Fathers	Increased risk	Oleary *et al*. [267]
			Children	NS	Wilson *et al*.; Oleary *et al*.; Van der Linden *et al*.; Doudney *et al*. [211,214,225,267]
				Increased risk	Pietrzyk *et al*.; Zhu *et al*.; Relton *et al*. [228,255,271]
			Mothers	NS	Wilson *et al*.; Relton *et al*.; Oleary *et al*. [211,228,267]
				Increased risk	Doolin *et al*.; Pietrzyk *et al*.; Zhu *et al*.; Van der Linden *et al*.; Candito *et al*.; Ouyang *et al*. *, Franke *et al*. [37,212,217,255,271,272,273]

* This reference is a meta-analyse; NS: no significant association found.

**Figure 4:**
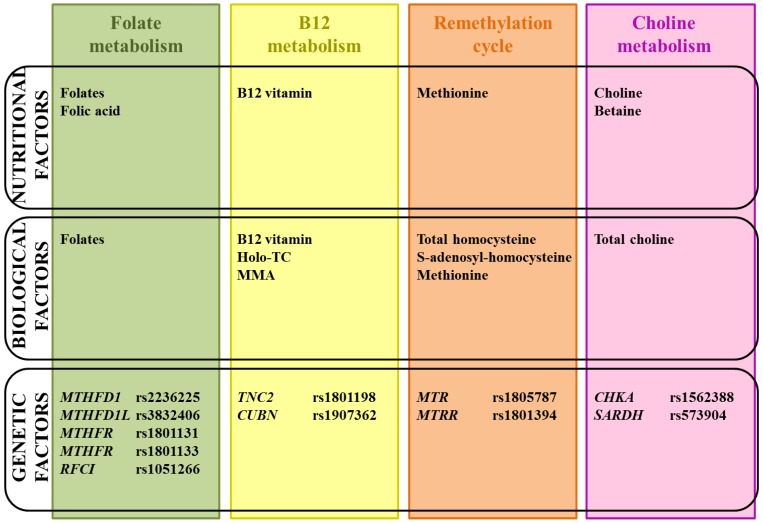
Nutritional, biological, and genetic risk factors of folate, B12 vitamin, remethylation and choline metabolisms that have (potentially) been related with NTDs risk. MMA: Methylmalonic acid, holo-TC: holo-transcobalamin.

Besides the role of folates in nucleotides biosynthesis, there are data pointing to the involvement of transmethylation by S-adenosylmethionine for the folic acid preventive effect as illustrated by methionine responsive mouse models. Choline, betaine and B12 metabolism, which is essential for optimal methylation, is emerging as a potential risk factor (Figure 4). The accumulation of scientific evidence warrants intervention studies based on periconceptional supplementation of vitamin B12 [274], choline or betaine [275] in the prevention of NTDs. Due to the multifactorial nature of the etiology of sporadic NTDs. The interpretation of these data will be challenging and large cohorts are needed to observe a potential effect. This underlines the need for international collaborations. Intervention studies are now needed to affirm or refute the hypothesis that choline, betaine or vitamin B12 supplementation has a role in the prevention of NTDs.
